# Comparison of performance of specific (SLEQOL) and generic (SF36) health-related quality of life questionnaires and their associations with disease status of systemic lupus erythematosus: a longitudinal study

**DOI:** 10.1186/s13075-020-2095-4

**Published:** 2020-01-10

**Authors:** Worawit Louthrenoo, Nuntana Kasitanon, Eric Morand, Rangi Kandane-Rathnayake

**Affiliations:** 1 Division of Rheumatology, Department of Internal Medicine, Faculty of Medicine, Chiang Mai University, Chiang Mai, 50200 Thailand; 2 School of Clinical Sciences at Monash Health, Monash University, Melbourne, Victoria Australia

**Keywords:** SLEQOL, SF36, Health-related quality of life, Patient-reported outcomes, Lupus, Disease activity, Low disease activity

## Abstract

**Background:**

The utility of generic health-related quality of life (HRQoL) questionnaires in patients with systemic lupus erythematosus (SLE) is uncertain. We compared the performance of generic (SF36) and specific (SLEQOL) HRQoL surveys by examining their associations with the Global Rating of Change (GRC) and SLE clinical indicators.

**Methods:**

The study included SLE patients who attended a single-center rheumatology clinic between 2013 and 2017. Patients completed both specific (SLEQOL) and generic (SF36) surveys and rated their GRC compared to the previous visit using a 7-point Likert scale on the same day of routine visits. Based on GRC scores, patients’ change in HRQoL was categorized as “no change,” “deterioration,” or “improvement.” Disease activity (SLEDAI-2K), flare, and lupus low disease activity state (LLDAS) were assessed at each visit, and organ damage (SDI) was determined annually. Pairwise correlations between SLEQOL and SF36 components were examined, and associations between GRC status and SLE disease indicators were compared using generalized estimating equations (GEE).

**Results:**

Three hundred thirty-seven patients with 2062 visits were included in the analysis. SLEQOL correlated significantly with SF36. Patients reported improvements in HRQoL in 58%, deterioration in 15%, and “no change” in 27% of all visits. Compared to the “no change” group, mean SF36 and SLEQOL scores were significantly lower in the deterioration group and higher in the improvement group. The magnitude of changes observed with SLEQOL and SF36 in the deterioration and improvement groups was similar. Patients in LLDAS had significantly higher mean scores in both SLEQOL and SF36. In contrast, patients with active disease, especially those with cutaneous, renal, central nervous system, and musculoskeletal activity, had significantly lower SLEQOL and SF36. Flare and organ damage were also associated with lower SLEQOL and SF36-PCS (physical component) but not with SF36-MCS (mental component).

**Conclusion:**

SLEQOL and SF36 similarly describe HRQoL in SLE. Both instruments demonstrated strong associations with GRC-based deterioration or improvement as well as SLE disease status. LLDAS was associated with improved HRQoL.

## Background

Systemic lupus erythematosus (SLE) is an autoimmune disease characterized by multiple organ involvement and a highly variable clinical course manifesting as recurrent relapses and exacerbations. Repeated and sustained inflammation of organ systems leads to organ dysfunction and permanent damage [1]. Due to a lack of effective therapy, patients suffer not only from inadequately controlled disease, but also from treatment-associated complications [2, 3]. All these factors contribute to significant increases in morbidity and mortality, and poor health-related quality of life (HRQoL) [1, 2], both of which are more pronounced in developing countries [4].

HRQoL is a multi-domain concept that evaluates patients’ overall perception of the impact of an illness and its treatment on his/her physical, emotional, and social function [5]. In recent years, HRQoL has gained more attention in SLE management where the focus has previously been on the control of disease activity and organ damage. In 2000, the Outcome Measures in Rheumatology Clinical Trials (OMERACT) group recommended that HRQoL assessments to be part of patient care [6]. SLE-specific HRQoL instruments have been developed and validated in several countries. These include the SLE Quality of Life (SLEQOL) [7–9], Lupus Quality of Life (LupusQoL) [10–12], Lupus Patient-Reported Outcome (LupusPRO) [13–17], SLE Symptom Checklist (SSC) [18, 19], and SLE Quality of Life Questionnaire (L-QoL) [20, 21]. In addition to these disease-specific HRQoL measures, clinicians and researchers have used generic HRQoL instruments such as the 36-item Short-Form Health Survey (SF36) [22, 23] and the EuroQoL-5D (EQ-5D) [24, 25]. While generic surveys have the advantage of allowing comparison with other disease states, disease-specific HRQoL surveys provide the opportunity to focus on SLE-specific issues, such as uncertainty of the course of the disease, side effects of treatment, and low self-esteem which are not captured by generic surveys [10]. Recommendations for HRQoL instrument used in research and clinical practice in SLE lack evidence that is based on a robust comparison between generic and disease-specific measures.

In this study, we compared the performance of specific and generic HRQoL instruments by assessing their sensitivity to change, defined by the Global Rating of Change (GRC). GRC is a HRQoL patient-reported outcome (PRO) measure in which patients rate their global health status compared to their previous visit and is thus designed to quantify patients’ improvement or deterioration over time. We used SLEQOL as the disease-specific HRQoL survey and SF36 as the generic HRQoL survey to perform this comparison. In addition, we examined the associations of SLEQOL and SF36 surveys with SLE clinical indicators such as SLE disease activity, organ damage, and lupus low disease activity state (LLDAS).

### Patients and methods

Adult, consenting SLE participants who attended the rheumatology clinic at the Chiang Mai University Hospital, Thailand, between October 2013 and June 2017 were recruited for this study. All patients met either the 1997 American College of Rheumatology Classification Criteria for Systemic Lupus Erythematosus [26] or the 2012 Systemic Lupus International Collaborating Clinics classification criteria for systemic lupus erythematosus [27]. Data were collected prospectively. SLEQOL was developed in Singapore and incorporates questions suitable for oriental cultures [7]. Both SLEQOL and SF36 surveys have been translated into Thai, validated [28, 29], and used in many clinical studies in Thailand [30, 31].

Patients completed SLEQOL and SF36 (version 2.0) surveys, and rated their GRC, at three to six monthly routine visits. Disease indicators including SLE disease activity, physician global assessment (PGA) of disease activity, and flare were captured at routine visits, and irreversible organ damage was captured annually. SLE disease activity was determined using the SLE Disease Activity Index 2000 (SLEDAI-2K) [32]. PGA was determined using a 10-cm visual analog scale (VAS) with a score of 0, 1, 2–2.5, and 3 corresponding to no, mild, moderately severe, and severe or life-threatening lupus disease activity, respectively [33]. Flare was determined using the SLE Flare Index (SFI) [34], and organ damage accrual was determined using the Systemic Lupus International Collaborating Clinics/American College of Rheumatology Damage Index (SDI) [35]. Active disease was defined as SLEDAI-2K > 4. The presence of organ damage was defined as SDI > 0. Attainment of lupus low disease activity state (LLDAS) at each visit was determined as published by Franklyn et al. [36]. We determined organ-specific disease activity using SLEDAI organ domains, based on at least one clinical feature in the proceeding 30 days as follows: CNS+ve (central nervous system) = seizure/psychosis/organic brain syndrome/visual disturbance/cranial nerve disorder/lupus headache/cerebrovascular activity (CVA); VAS+ve = vasculitis; MSK+ve = arthritis/myositis; renal+ve = proteinuria/hematuria/pyuria/urinary casts; cutaneous+ve = rash/alopecia/mucosal ulcers; and serological+ve = low complement or/and increased DNA binding activity.

The SLEQOL survey consists of 40 items that fall into 6 domains: physical functioning, activities, symptoms, treatment, mood, and self-image [28]. Each item has a 7-point scale ranging from 1 (“not difficult at all,” “no trouble at all,” or “not often at all”) to 7 (“extremely difficult,” “extremely problem at all,” or “extremely often”). The sum of the scores ranges from 40 to 280, where high scores indicate poor HRQoL. The S36 survey consists of 36 items that fall into 8 domains: physical functioning (PF), role physical (RP), role emotional (RE), social functioning (SF), mental health (MH), energy/vitality (VT), body pain (BP), and general health (GH) perception [29]. Each item that has a 5-point scale ranging from 1 (“best,” “not difficult at all,” “no trouble at all,” or “not often at all”) to 5 (“worst,” “extremely difficult,” “extremely problem at all,” or “extremely often”). The final domain scores were derived using the QualityMetric Health Outcomes Scoring Software 5.0 (Optum, Lincoln, RI, USA) through which scores were transformed to 0–100, where low scores indicate poor HRQoL. In addition to the domain scores, 2 summary scores, physical component summary (PCS) and mental component summary (MCS), were derived and normalized against the US population.

We used the processes of re-scoring and standardization [37] to compare SLEQOL with SF36 results. Each SLEQOL item was re-scored by subtracting the original score from 8 and subsequently re-scaled using the following linear equation:

$$ Y=1+\left(\mathrm{SLEQoL}\ \mathrm{Original}\ \mathrm{score}-A\right)\times \frac{\left(100-1\right)}{\left(B-A\right)},\kern0.5em \mathrm{where}\ A=\min .\kern0.5em \mathrm{score}\ \mathrm{and}\kern0.5em B=\max .\kern0.5em \mathrm{score} $$

Few questions had missing values, and we adopted the half-mean imputation method where the missing scores were replaced by the half-mean of the corresponding domain [37].

Patients rated GRC in HRQoL compared to the previous visit using a 7-point Likert scale (from − 7 [a very great deal worse] to + 7 [a very great deal better]). Based on the GRC scores, patients were grouped into either “no change” (− 1 to + 1), “deterioration” (− 2 to − 7), or “improvement” (+ 2 to + 7) categories [38].

This study was approved by the Faculty of Medicine Human Research Ethics Committee, Chiang Mai University.

### Statistical analysis

Statistical analyses were performed using Stata version 15.1 (StataCorp, College Station, TX, USA). Continuous variables were summarized as median (interquartile range [IQR], range), and categorical variables were described as frequency (%). Time-adjusted means (TAMs) were calculated for both SLEQOL and SF36 domain scores to estimate the average values accounting for varying time intervals between visits. Similarly, TAM SLEDAI-2K was derived to estimate the average values accounting for varying time intervals between visits [39].

Correlations among TAMs of SLEQOL and SF36 domains were examined using Pearson pair-wise correlation coefficients. The generalized estimating equations (GEE) method was used to examine the associations of GRC categories and SLE clinical indicators (SLEDAI-2K > 4, flare, organ damage, and LLDAS) with SLEQOL and SF36 surveys. SLEQOL/SF36 results were analyzed as the dependent variable throughout the analysis. We also examined the associations between GRC categories and clinical indicators in which clinical indicators were treated as the outcomes. For SLEQOL and SF36 survey outcome assessment, we specified Gaussian distribution for the family along with an identity link, and for the assessment of clinical indicators as outcomes, we specified binomial distribution with a logit link. We used an exchangeable correlation matrix in all models. Robust standard errors were derived adjusting for patient clustering. Demographic variables with *p* values < 0.1 in univariable GEE analyses were included in multivariable models to investigate independent associations of clinical indicators with HRQoL after adjustment for confounders. LLDAS exhibits strong negative collinearity with SLEDAI-2K and flare; therefore, separate multivariable GEE models were carried. Results were reported as either mean change (regression coefficients) or odds ratios with corresponding 95% confidence interval (95% CI). Positive mean change indicates higher/improved HRQoL whereas negative mean change indicates poorer/worse HRQoL. A *p* value < 0.05 was considered statistically significant.

## Results

The study included 337 SLE patients and a total of 2062 visits. Data on SF36 and SLEQOL were available from 2057 and 2058 visits, respectively. There were 17 patients with baseline visit data only, and therefore, GRC was not rated. As GRC measures inter-visit change, it was available on 1728 visits. HRQoL instruments were completed with a median ([IQR] (range)) of 7 ([4, 8] (1, 9)) time per patient and GRC with a median of 6 ([3, 7] (0, 8)) times.

### Patient characteristics

Table 1 presents a summary of patient characteristics. In brief, approximately 96% of patients were female, with a median ([IQR] (range)) age at enrollment of 37 ([28, 48] (18, 74)) years and a median disease duration of 7 ([3, 13] (0, 36)) years. Approximately 7% had a family history of SLE, and 47% had tertiary-level education. Patients were observed for a median of 3.2 ([1.6, 3.4] (0, 4.3)) years. About 95% of patients were treated with glucocorticoids, with a TAM prednisolone dose across the period of observation of 5.8 ([3.7, 9.3] (0, 61)) mg/day. In addition, 84% of patients had received immunosuppressive drugs and 38% used anti-malarial drugs during the observation period. The median TAM SLEDAI-2K was 3.5 ([2.0, 5.6] (0, 20)). About 56% of patients experienced a flare, and 52% of patients had irreversible organ damage. Approximately 81% of patients achieved LLDAS at least once.

**Table 1:** Patient demographics and disease characteristics

	*n* = 337, median [IQR] (range) or *n* (%)
Demographics	
Age at enrolment (years)	37 [28, 48] (18, 74)
Age at diagnosis (years)	26 [19, 38] (1, 69)
Disease duration (years)	7 [3, 13] (0, 36)
Study duration (years)	3.2 [1.6, 3.4] (0, 4.3)
Female	325 (96.4%)
Current smoker at enrolment	3 (0.9%)
Family history of SLE	25 (7.4%)
Education level	
Primary	83 (24.8%)
Secondary	96 (28.7%)
Tertiary	156 (46.6%)
Medications use	
Prednisolone ever	319 (94.7%)
TAM Prednisolone dose	5.8 [3.7, 9.3] (0, 61)
Anti-malarials ever	129 (38.3%)
Immunosuppressants ever	284 (84.3%)
Clinical indicators	
TAM PGA	0.4 [0.3, 0.7] (0.2, 1.9)
TAM SLEDAI-2K	3.5 [2.0, 5.6] (0, 20)
Organ damage score	1 [0, 1] (0, 6)
Flare (mild/moderate/severe) ever	189 (56.1%)
Organ damage present	176 (52.2%)
Achieved LLDAS ever	272 (80.7%)

Summary statistics of individual domains of SLEQOL and SF36 surveys are presented in Additional file 5: Table S1. The TAM SLEQOL total score of the study population was 89.8 ([81.7, 94.9] (1, 100)), and the TAM physical component summary (PCS) and TAM mental component summary (MCS) of the SF36 survey were 46.8 ([42.0, 52.1] (17.6, 60.2)) and 49.4 ([42.9, 55.0] (20.5, 63.8)), respectively. Overall, SLEQOL domains scored slightly better than SF36. In addition, based on GRC ratings, 84% of patients reported improvement and about 40% of patients reported deterioration at least once. Univariable GEE associations of patient demographics with HRQoL surveys (SLEQOL/SF36) are shown in Additional file 6: Table S2. Older age at SLE diagnosis was associated with lower SF36-PCS scores but not SF36-MCS or SLEQOL scores. Longer study duration was associated with higher SF36-MCS scores; each year of study duration was associated with an increase of MCS score of 1.30 (95% CI 0.52, 2.07, *p* < 0.01). Patients with higher education levels had significantly higher scores in both SLEQOL and SF36 surveys. Other demographics including gender and SLE family history were not associated with HRQoL (Additional file 6: Table S2).

### Correlation between SLEQOL and SF36

**Fig. 1:**
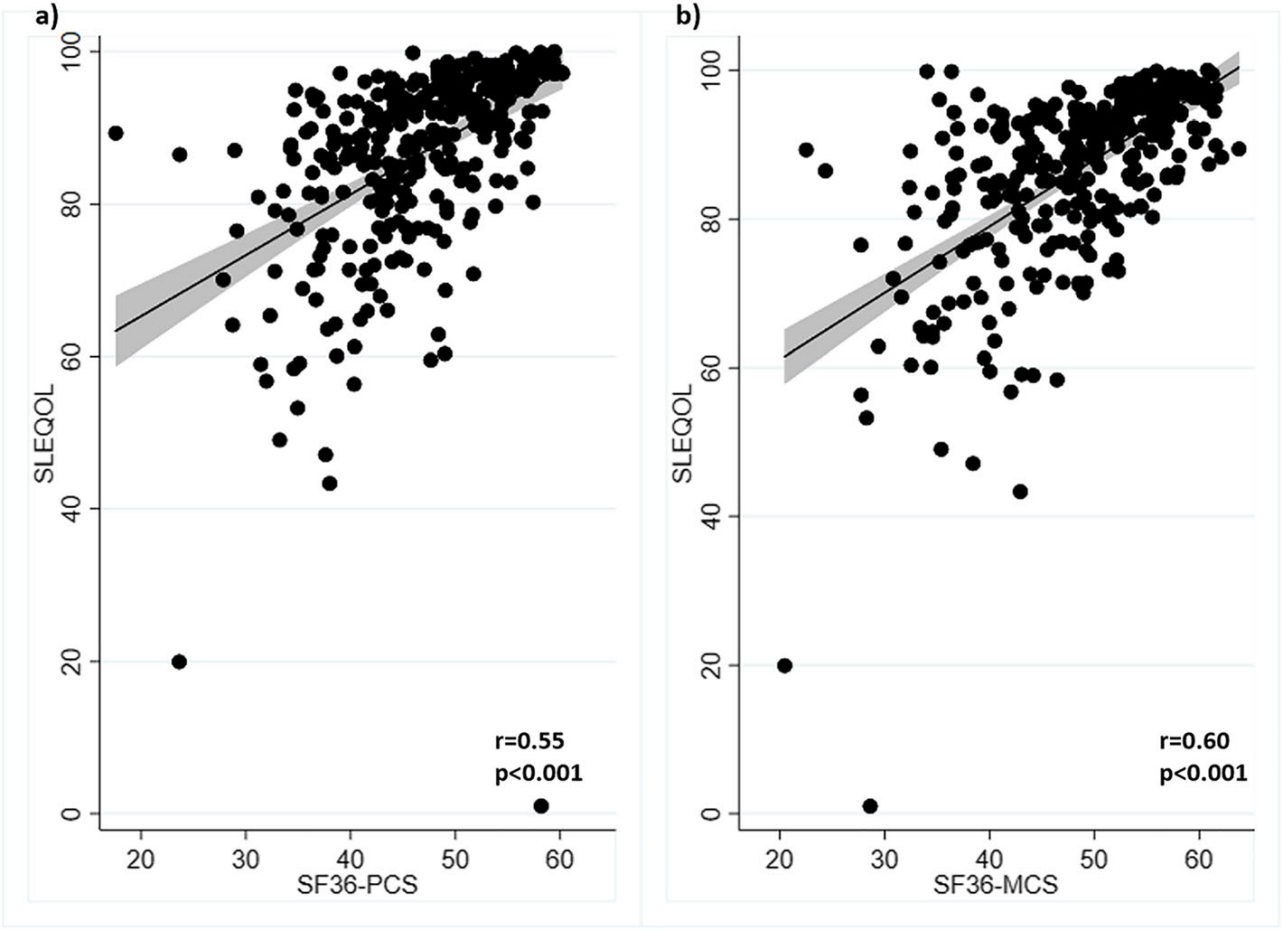
Scatterplots of SLEQOL with **a** SF36 PCS and **b** SF36 MCS scores

**Fig. 2:**
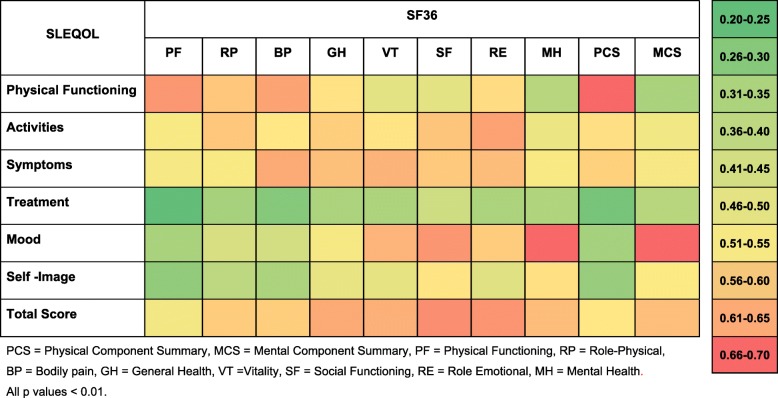
Heat map of pairwise correlation coefficients among SLEQOL and SF36 components. Time-adjusted means (TAMs) of each component were used to calculate correlation coefficients

Pairwise correlations among different components of the two surveys were determined. Correlations between the TAM total SLEQOL score and the SF36 survey PCS and MCS scores were moderate (*r* = 0.55 and 0.60, respectively; *p* values < 0.001) (Fig. 1). Individual SLEQOL survey domains correlated with SF36 survey domains positively and significantly (all *p* values < 0.01) at varying strength (Fig. 2). Strong correlations were observed between physical domains of SLEQOL and SF36 physical activity indicators PCS, physical function, and bodily pain domains. Similarly, strong correlations were observed between mental health domains of SLEQOL and SF36 MCS and other mental health components including mental health and role emotional. The strongest correlation was between SLEQOL-mood and SF36-mental health (*r* = 0.70, *p* < 0.001). Moreover, SLEQOL-symptoms correlated well with SF36-bodily pain, vitality, and role emotion domains. SLEQOL-treatment domain demonstrated the weakest correlations with SF36 survey domains (Fig. 2).

### Global Rating of Change

The majority of patients reported at least 2 different GRC categories (no change, improvement, or deterioration) between visits across the study period; 74 reported each of the 3 categories at various times (Additional file 1: Figure S1a). In contrast, 6 and 15 patients respectively reported deterioration or no change at all visits, while 64 patients reported improvement at all visits. On per visit basis, improvement was reported at 58.3% of visits, no change in 27.3%, and deterioration at 14.4% of visits (Additional file 1: Figure S1b).

To evaluate associations of patient-reported GRC with clinical status, we investigated the distribution of patient characteristics and clinical features across GRC categories (Additional file 7: Table S3). Demographics including age (both at enrolment and at diagnosis), gender, smoking status, family history, and education level did not differ among the groups. The six patients who reported deterioration at every visit had the shortest study duration with a median (IQR) of 5.8 (5.5, 6.9) months; in contrast, patients who reported all three categories had the longest study duration with a median follow-up of 40 (38, 44) months. Patients who reported deterioration at all visits had the highest TAM SLEDAI-2K, received the highest doses of prednisolone, and had the least proportion of time in LLDAS.

### Associations of GRC categories with SLEQOL, SF36, and clinical indicators

To determine whether patient-reported GRC correlated with changes in instrument-measured HRQoL, we examined the magnitude of mean changes in SLEQOL and SF36 scores in GRC categories. The overall HRQoL survey results and clinical indicators are presented in Table 2, and domain-specific HRQoL results are presented in Additional file 8: Table S4. Mean scores of SLEQOL and SF36 overall and domain-specific scores in the deterioration group were significantly lower compared to the no change group. In contrast, in the improvement group, SF36-PCS scores were significantly higher compared to the no change group. SF36 bodily pain, general health, and vitality scores were also significantly higher in the improvement group than the no change group, as was the SLEQOL-treatment domain. Mean changes in other SLEQOL domains did not reach statistical significance (Additional file 8: Table S4).

**Table 2:** Univariable GEE associations of global rating of change status with SLEQOL, SF36 surveys and clinical indicators

GRC status	Outcomes						
	HRQoL surveys			SLE clinical indicators (present at visit)			
	SLEQOL (SLE-specific)	SF36 (generic)		In LLDAS	SLEDAI-2K > 4	MM/S flare	Organ damage
		PCS	MCS				
	Mean (95% CI)	Mean (95% CI)	Mean (95% CI)	Freq. visits (%)	Freq. visits (%)	Freq. visits (%)	Freq. visits (%)
No change (no. visits = 471)	88.4 (87.2, 89.7)	46.9 (46.1, 47.8)	49.5 (48.6, 50.5)	219 (46.6%)	127 (27.0%)	82 (17.4%)	215 (45.6%)
Deterioration (no. visits = 250)	79.6 (77.4, 81.7)	42.8 (41.8, 43.8)	47 (45.7, 48.2)	82 (32.8%)	97 (38.8%)	66 (26.4%)	157 (62.8%)
Improvement (no. visits = 1007)	88.8 (87.7, 89.9)	47.9 (47.2, 48.7)	50.1 (49.2, 50.9)	525 (52.2%)	259 (25.7%)	130 (12.9%)	517 (51.3%)
	Mean diff. (95% CI), *p* value	Mean diff. (95% CI), *p* value	Mean diff. (95% CI), *p* value	Odds ratio (95% CI), *p* value	Odds ratio (95% CI), *p* value	Odds ratio (95% CI), *p* value	Odds ratio (95% CI), *p* value
No change (ref.)	0	0	0	1.00	1.00	1.00	1.00
Deterioration	− 8.9 (−11.1, − 6.7), *p* < 0.01	− 4.12 (− 5.2, − 3.1), *p* < 0.01	− 2.53 (− 3.9, − 1.2), *p* < 0.01	0.59 (0.43, 0.80), *p* < 0.01	1.61 (1.18, 2.19), *p* < 0.01	1.83 (1.27, 2.64), *p* < 0.01	1.12 (1.01, 1.25), *p* = 0.04
Improvement	0.4 (− 0.5, 1.3), *p* = 0.4	0.96 (0.3,1.6), *p* < 0.01	0.54 (− 0.2,1.3), *p* = 0.17	1.30 (1.07, 1.57), *p* = 0.01	0.85 (0.70, 1.04), *p* = 0.11	0.70 (0.52, 0.95), *p* = 0.02	1.01 (0.93, 1.10), *p* = 0.8

*Freq. visits* = frequency of visits, *Mean diff.* = mean difference

Significant associations of GRC status with clinical indicators were observed (Table 2). Patients who reported deterioration were 41% less likely to be in LLDAS whereas those who reported improvement were 30% more likely to be in LLDAS. Patients who reported deterioration were also significantly more likely to have active diseases, flare, and organ damage (Table 2).

### Associations of SLE disease status indicators with HRQoL measures

We next examined the longitudinal associations between clinical indicators and HRQoL (Tables 3 and 4). SLEQOL scores were significantly higher among patients in LLDAS, and correspondingly lower in patients with SLEDAI-2K > 4, flare, or organ damage, in univariable GEE analysis (Table 3). Patients in LLDAS scored significantly higher scores in all six domains (physical functioning, activities, symptoms, treatment, mood, and self-image) of SLEQOL compared to those who were not (Additional file 2: Figure S2a), while the presence of active disease had the reverse associations (Additional file 2: Figure S2c). The analysis of organ-specific disease activity revealed that central nervous system (CNS), vasculitis, musculoskeletal, renal, and cutaneous SLEDAI-2K domains were significantly associated with poorer SLEQOL (Additional file 3: Figure S3). Flares were associated with physical functioning, activities, symptoms, and mood but not treatment or self-image domains (Additional file 2: Figure S2e). Similarly, organ damage was associated with physical functioning, activities, and symptoms but was not associated with patients’ treatment, self-image, or mood (Additional file 2: Figure S2g).

**Table 3:** Univariable GEE, longitudinal associations of SLE clinical indicators with SLEQOL and SF36 survey scores

	HRQoL survey outcomes					
	SLEQOL-total score		SF36-PCS		SF36-MCS	
	Mean (95% CI)	RC (95% CI), *p* value	Mean (95% CI)	RC (95% CI), *p* value	Mean (95% CI)	RC (95% CI), *p* value
LLDAS status						
Not in LLDAS	85.7 (84.4, 86.9)	Ref.	46.2 (45.4, 47.0)	Ref.	48.1 (47.2, 48.9)	Ref.
In LLDAS	88.7 (87.6, 89.9)	3.0 (2.0, 4.0), *p* < 0.01	47.7 (46.9, 48.5)	1.5 (0.9, 2.1), *p* < 0.01	49.7 (48.8, 50.6)	1.6 (0.9, 2.4), *p* < 0.01
SLEDAI-2K						
SLEDAI-2K ≤ 4	88.0 (86.9, 89.2)	Ref.	47.4 (46.7, 48.2)	Ref.	49.2 (48.4, 50.0)	Ref.
SLEDAI-2K > 4	84.8 (83.4, 86.3)	− 3.2 (− 4.4, − 2.0), *p* < 0.01	45.6 (44.6, 46.6)	− 1.8 (− 2.6, − 1.0), *p* < 0.01	47.8 (46.9, 48.8)	− 1.4 (− 2.2, − 0.5), *p* < 0.01
Flare						
Absent	87.4 (86.2, 88.5)	Ref.	47.2 (46.5, 47.9)	Ref.	48.9 (48.1, 49.8)	Ref.
Present	85.7 (84.1, 87.3)	− 1.7 (− 2.8, − 0.5), *p* < 0.01	45.2 (44.2, 46.2)	− 2.0 (− 2.6, − 1.3), *p* < 0.01	48.3 (47.3, 49.4)	− 0.6 (− 1.4, 0.2), *p* = 0.15
Organ damage						
Absent	88.4 (87.0, 89.8)	Ref.	48.5 (47.7, 49.4)	Ref.	48.8 (47.8, 49.8)	Ref.
Present	85.8 (84.2, 87.3)	− 2.6 (− 4.5, − .08), *p* < 0.01	45.3 (44.3, 46.3)	− 3.2 (− 4.4, − 2.1), *p* < 0.01	48.8 (47.7, 49.9)	0.0 (− 1.4, 1.4), *p* = 0.9

RC = regression coefficient = mean difference

**Table 4:** Multivariable, longitudinal associations of SLE clinical indicators with SLEQOL and SF36 survey scores

	HRQoL survey outcomes		
	SLEQOL-total score, RC* (95% CI), *p* value	SF36-PCS, RC* (95% CI), *p* value	SF36-MCS, RC* (95% CI), *p* value
In LLDAS	3.0^1^ (2.1, 4.1), *p* < 0.001	1.5^1^ (1.0, 2.1), *p* < 0.001	1.6^1^ (0.9, 2.4), *p* < 0.001
SLEDAI-2K > 4	− 3.12^2^ (− 4.44, − 1.79), *p* < 0.01	− 1.52^3^ (− 2.37, − 0.68), *p* < 0.01	− 1.36^4^ (− 2.18, − 0.54), *p* < 0.001
Flare	− 0.58^5^ (− 1.83, 0.66), *p* = 0.4	− 1.50^6^ (− 2.20, − 0.80), *p* < 0.01	–
Organ damage	− 2.53^7^ (− 4.33, − 0.74), *p* = 0.01	− 2.66^8^ (− 3.72, − 1.59), *p* < 0.01	–

RC* = regression coefficient = mean difference compared to patients without clinical indicators

^1^LLDAS associations adjusted for education level. SLEDAI-2K associations adjusted for ^2^flare, organ damage, and education level; ^3^flare, organ damage, education level, and age at enrolment; ^4^disease duration and education level. Flare associations adjusted for ^5^SLEDAI-2K > 4, organ damage, and education level, and ^6^SLEDAI-2K > 4, organ damage, education level, and age at enrolment. Organ damage associations adjusted for ^7^flare, SLEDAI-2K > 4, and education level, and ^8^SLEDAI-2K > 4, flare, organ damage, education level, and age at enrolment

Similar to the findings with SLEQOL, SF36-PCS were statistically significant associated with LLDAS, SLEDAI-2K > 4, flare, and damage accrual in univariable GEE analysis (Table 3). Patients in LLDAS had significantly higher mean SF36-PCS and SF36-MCS scores whereas patients with active disease had significantly lower mean SF36-PCS and SF36-MCS (Table 3**,** Additional file 2: Figure S2b and d). Flare and organ damage were also significantly associated with lower SF36-PCS but not with SF36-MCS scores (Table 3, Additional file 2: Figure S2f and h). The analysis of organ-specific disease activity indicated that CNS, vasculitis, musculoskeletal, renal, and cutaneous SLEDAI-2K domain activities were significantly associated with poorer SF36 domain scores (Additional file 4: Figure S4)*.*

We performed separate multivariable analyses for LLDAS and other disease indicators due to strong inverse collinearity between LLDAS and SLEDAI-2K or flare. LLDAS remained significantly associated with better SLEQOL, SF36-PCS, and SF36-MCS scores (Table 4). Similarly, active disease remained strongly negatively associated with SLEQOL, SF36-PCS, and SF36-MCS (Table 4), organ damage remained strongly associated with reduced SLEQOL and SF36-PCS scores, and flare remained significantly associated with lower SF36-PCS score but attenuated its association with SLEQOL (Table 4). All the observed associations with statistical significance are summarized in Additional file 9: Table S5.

## Discussion

The assessment of HRQoL in SLE continues to attract attention, based on the emerging understanding that physician and laboratory measures do not capture all information important to patients [40]. Multiple instruments have been developed for SLE alongside well-validated generic HRQoL instruments, but the comparative utility of generic and SLE-specific instruments remains unclear. In this prospective longitudinal study, we observed significant correlations between the SLE-specific (SLEQOL) and generic (SF36) instruments, and comparable associations in terms of their sensitivity to change as assessed using the Global Rating of Change (GRC). A simple GRC report of deterioration was associated with worse clinical indicators and HRQoL. While the presence of active disease, flare, and organ damage was significantly associated with poor HRQoL, as assessed using both generic and SLE-specific instruments, LLDAS was significantly associated with better HRQoL.

To our knowledge, this is the longest observational study to compare the associations of SLEQOL and SF36 instruments with patients’ GRC status and SLE clinical indicators. GRC scales are designed to quantify a patient’s impression of improvement or deterioration in HRQoL over time, either to determine an intervention effect or monitor the clinical course of a disease [41]. Although patients reported deterioration in only 15% of visits, the association of GRC deterioration with poor quality of life, assessed using both SLEQOL and SF36, was significantly more prominent than the association of GRC improvements with better HRQoL scores. Recently, McElhone et al. reported similar relationships between a disease-specific instrument (LupusPRO) and SF36 in relation to GRC status in a 10-month study [42]. In contrast to our study, they found a greater magnitude of change in those who reported improvement than in those reporting deterioration.

Several previous cross-sectional studies have shown varying degrees of correlation between SLEQOL and SF36. Aziz et al. recently reported strong correlations between SLEQOL and SF36-PCS and SF36-MCS [8], including strong correlations between SLEQOL-physical functioning component and SF36-PCS, as well as between SLEQOL-mood and SF36-MCS [8]. We observed similar relationships between these domains in the current, longitudinal study, with the strongest correlation observed between SLEQOL-mood and SF36-mental health. In another cross-sectional study, Jiang et al. reported weak to moderate correlations between SLEQOL and SF36 [9]. Leong et al., the research group who developed the SLEQOL, also found weak correlations between SLEQOL and SF36 domains [7].

Our findings suggest that HRQoL assessed using a generic PRO yields similar information to that captured using an SLE-specific HRQoL instrument. A few previous studies have compared generic HRQoL PROs with other SLE-specific PROs. For instance, McElhone et al. [10] compared LupusQoL with SF36 in a cross-sectional study and found strong correlations among physical health/physical functioning, emotional health/mental health, pain/bodily pain, and fatigue/vitality domains. Jolly et al. [13] compared the LupusPRO and SF36 surveys in a cross-sectional study and found moderate to strong correlations among various domains: the strongest correlation was observed between the LupusPRO pain-vitality domain and the bodily and vitality domains of the SF36. In our study, SLEQOL total, physical functioning, activities, and symptoms scores also correlated well with most SF36 domains. All these studies suggest that the use of generic PROs to assess HRQoL in SLE patients is broadly acceptable. As generic PROs provide the opportunity to assess HRQoL in one disease against other diseases, for example, SLE vs. rheumatoid arthritis or ankylosing spondylitis [43, 44], this further strengthens the case for the use of generic HRQoL instruments in SLE.

One exception is that the SLEQOL Treatment domain correlated poorly with SF36 components. This perhaps indicates that generic surveys may not capture HRQoL issues that relate to medications specific to diseases. This was in line with previous observations reported by McElhone et al. [10] in which the authors used the LupusQoL as the specific SLE HRQoL instrument, and compared the results with SF36. In some circumstances, it could be beneficial to assess HRQoL using both generic and disease-specific instruments. However, conducting routine surveys may not be suitable for all clinical settings as this is time and resource intensive.

We observed that active disease, especially in CNS, cutaneous, and musculoskeletal domains, was significantly associated with poor HRQoL. In addition, the presence of flare or organ damage at visits was associated with significantly lower mean scores of SLEQoL and SF36-PCS, but not SF36-MCS. Similar associations between disease activity and organ damage and SF36-PCS and MCS were recently reported by Golder et al. in a large multi-center cross-sectional study [45]. In the same study, the authors reported the association of LLDAS with better HRQoL assessed using SF36 survey in a cross-sectional study. We here confirm that LLDAS is associated with better HRQoL using an SLE-specific instrument. Two very recent studies in the USA and Latin America have demonstrated longitudinal associations between LLDAS and improved HRQoL assessed using the generic SF36 as well as the SLE-specific LupusQoL survey [46, 47]. This observed association between LLDAS and improved HRQoL is important given LLDAS is a composite measure of both disease activity and treatment burden and an attainable target for SLE patient treatment.

Not all studies have shown associations between SLEQOL and disease activity and organ damage, potentially due to the differences in patient populations, variations in HRQoL instruments used, and discrepancies in the assessment of disease activity [48]. In the original study reporting the development of SLEQOL, Leong et al. found a negligible correlation between SLEQOL summary score and SLE disease activity and organ damage [7]. A study in China by Jiang et al. using the Chinese version of the SLEQOL also found negligible correlation between SLEQOL summary score and disease activity [9]. Aziz et al. reported moderate correlations of SLEQOL with disease activity and organ damage in a cross-sectional study conducted in Arabic SLE patients [8].

The correlation of SLE disease activity and damage with other disease-specific and generic HRQoL measures has been reported in several studies. Studies from the UK [10] and Italy [11] have reported strong correlations between LupusQoL and SF36, and most LupusQoL domains have been associated with SLE disease activity although not with organ damage. Studies also report comparable correlations between LupusPro and generic HRQoL (SF36 and/or EQ-5D) [13–16]. While some domains of LupusPro have found to be weakly or even negatively correlated with SLE disease activity and damage [13–16], other studies have also shown poor correlations between generic measures (EQ-5D and SF6D) and SLE disease indicators [25].

Limitations of this study include that it was performed at a single center. However, this study used a large cohort, followed prospectively, and used a longer period of follow-up than that the majority of studies reported. This study was performed in Thailand in the Thai language, but all the PROs used have been previously validated in Thai [28–31]. Finally, this was an observational study using usual care rather than an intervention; the comparability of generic vs. SLE-specific HRQoL instruments needs to be confirmed in the setting of an interventional trial with an effective agent.

Herein, we have demonstrated that while lupus-specific and generic HRQoL instruments have their own advantages and disadvantages, the use of either can be recommended in order to incorporate patient-reported information in medical decision-making during clinical practice and in trials. Even a simple, generic HRQoL instrument includes a minimum of physical, social, and emotional functioning domains, compared to which the advantage of specific HRQoL instruments which capture more disease- and treatment-specific features [49, 50] is limited. As simple HRQoL surveys are more widely validated in different languages and cultures, and have the advantage of allowing comparison of lupus with other diseases, the use of generic HRQoL tools in lupus research should be carefully considered.

## Conclusions

This study confirmed that both disease-specific (SLEQOL) and generic (SF36) instruments were correlated and performed similarly in the assessment of HRQoL in SLE patients. This study also provides a comprehensive analysis of patient-reported HRQoL outcomes in SLE, longitudinally captured for a median 3-year period. The magnitude of changes in SLEQOL and SF36 overall and component-specific scores was greatest in patients who self-reported deterioration in HRQoL. Patients in LLDAS had significantly better HRQoL than those who were not, while active disease, flare, and organ damage were associated with poorer HRQoL. Both SLEQOL and SF36 surveys were sensitive to change over time.

## Supplementary information

**Additional file 1: Figure S1.** Global Rating of Change (GRC) categories a) per patient basis, and b) per visit basis.

**Additional file 2: Figure S2.** Radar charts showing mean HROoL scores with 95% confidence intervals in SLE patients according to disease indicators. Figures a, c, e, and g are based on SLEQOL, and figures b, d, f and h are based on SF36. LLDAS = lupus low disease activity state, SLEDAI = systemic lupus erythematous disease activity index -2K.

**Additional file 3: Figure S3.** Radar charts showing mean SLEQOL scores with 95% confidence interval according to organ specific disease activity. SLEDAI = systemic lupus erythematosus disease activity index -2K, CNS = central nervous system, VAS = vasculitis, MSK = musculoskeletal, CUT = cutaneous, SEROL = serological.

**Additional file 4: Figure S4.** Radar charts showing mean SF36 scores with 95%confidence interval according to organ specific disease activity. SLEDAI = systemic lupus erythematosus disease activity index-2K, CNS = central nervous system, VAS = vasculitis, MSK = musculoskeletal, CUT = cutaneous, SEROL = serological.

**Additional file 5: Table S1.** Summary of time-adjusted mean scores of health related quality of life indicators.

**Additional file 6: Table S2.** Univariable GEE associations of patient demographics with health-related quality of life surveys.

**Additional file 7: Table S3.** Patient characteristics by GRC categories.

**Additional file 8: Table S4.** Associations of GRC categories with SLEQOL and SF36 domains.

**Additional file 9: Table S5.** Summary of statistically significant associations between SLE clinical indicators and HRQoL surveys.

## Data Availability

The datasets used and/or analyzed in this study are available from the corresponding author on reasonable request.

## Abbreviations

BP: Bodily pain
CNS: Central nervous system
EQ-5D: EuroQoL-5D
GEE: Generalized estimating equations
GH: General health
GRC: Global Rating of Change
HRQoL: Health-related quality of life
IQR: Interquartile range
LLDAS: Lupus low disease activity state
L-QoL: SLE Quality of Life Questionnaire
LupusPRO: Lupus Patient-Reported Outcome
MCS: Mental component summary
MH: Mental health
OMERACT: Outcome Measures in Rheumatology Clinical Trials
PCS: Physical component summary
PF: Physical functioning
PGA: Physician Global Assessment
PRO: Patient-reported outcome
RE: Role emotional
RP: Role physical
SDI: Systemic Lupus International Collaborating Clinics/American College of Rheumatology Damage Index
SF: Social functioning
SF36: 36-Item Short-Form Health Survey
SFI: SLE Flare Index
SLE: Systemic lupus erythematosus
SSC: SLE Symptom Checklist
SLEDAI-2K: SLE Disease Activity Index 2000
SLEQOL: SLE Quality of Life
TAM: Time-adjusted means
VT: Vitality
95% CI: 95% confidence interval
