# Supplementary figures and images for: Comparison of performance of specific (SLEQOL) and generic (SF36) health-related quality of life questionnaires and their associations with disease status of systemic lupus erythematosus: a longitudinal study
Source: Arthritis Res Ther. 2020 Jan 10;22:8. doi: 10.1186/s13075-020-2095-4 (PMC6954627; doi:10.1186/s13075-020-2095-4)

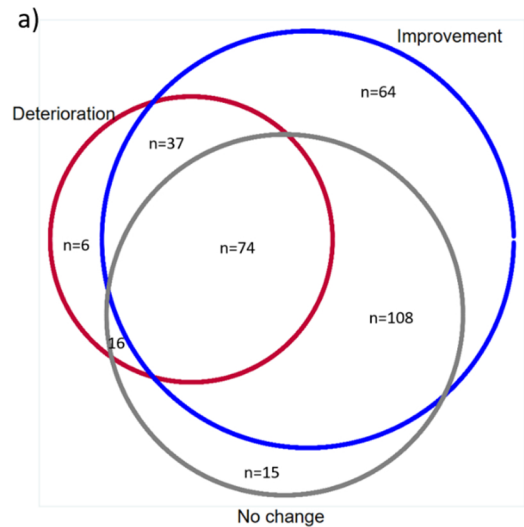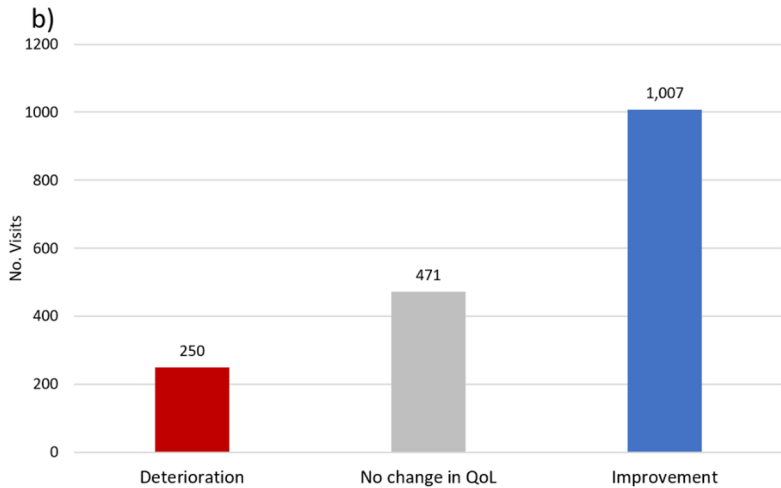

Supplement: Supplementary file 1 — Additional file 1: Figure S1. Global Rating of Change (GRC) categories a) per patient basis, and b) per visit basis. [file 13075_2020_2095_MOESM1_ESM.pdf]

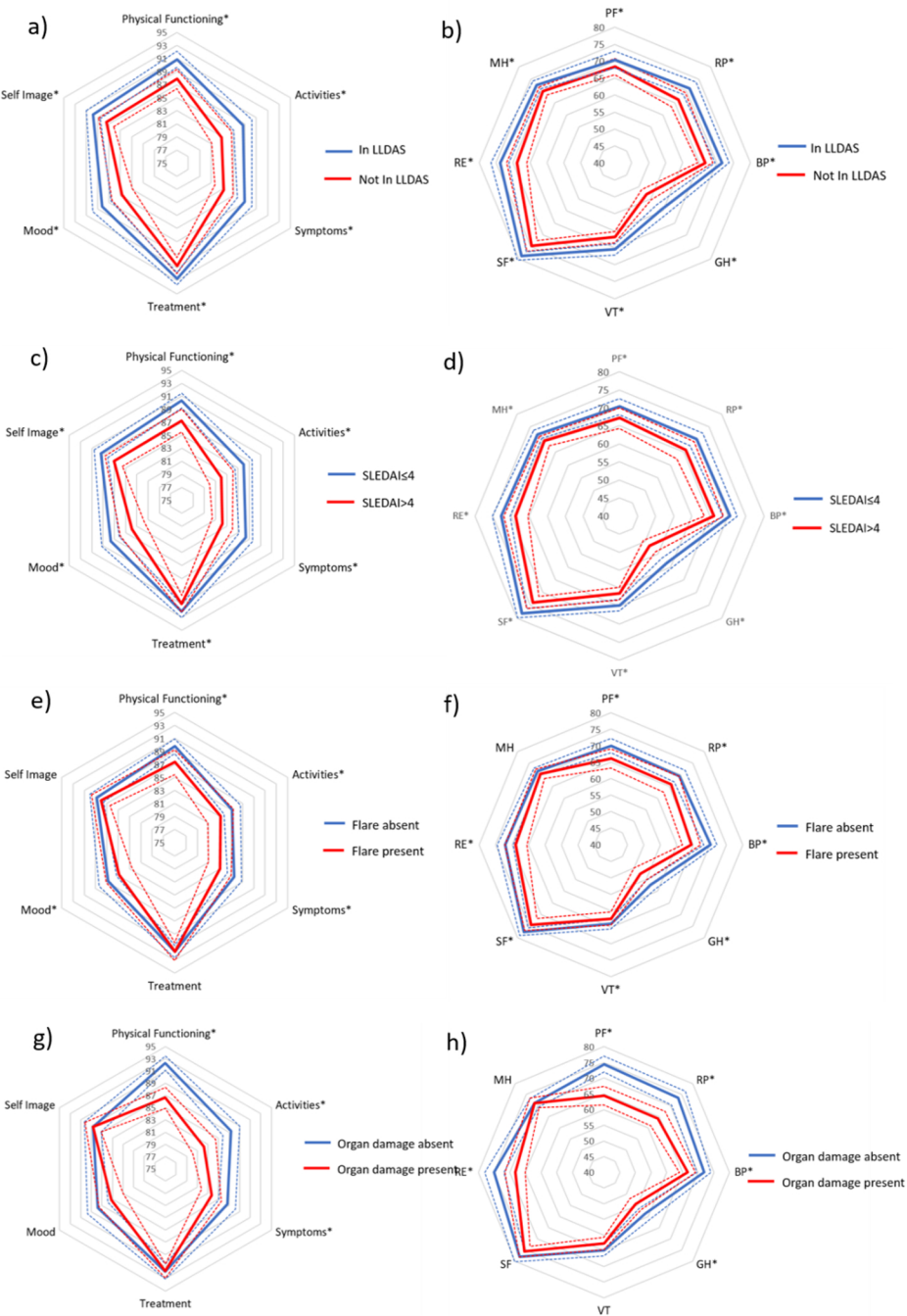

Supplement: Supplementary file 2 — Additional file 2: Figure S2. Radar charts showing mean HROoL scores with 95% confidence intervals in SLE patients according to disease indicators. Figures a, c, e, and g are based on SLEQOL, and figures b, d, f and h are based on SF36. LLDAS = lupus low disease activity state, SLEDAI = systemic lupus erythematous disease activity index -2K. [file 13075_2020_2095_MOESM2_ESM.pdf]

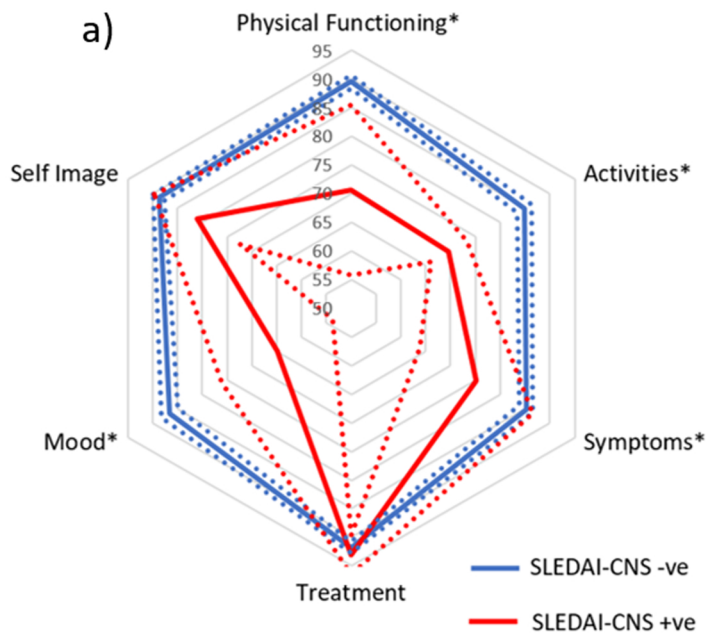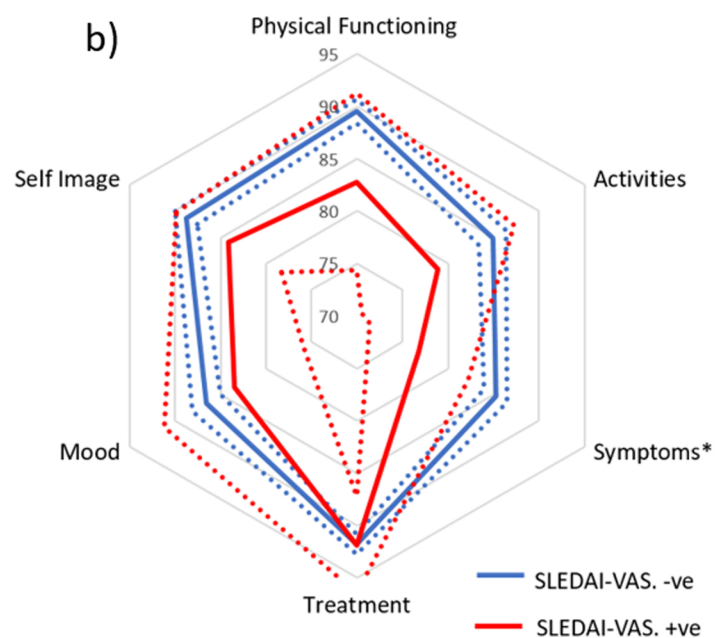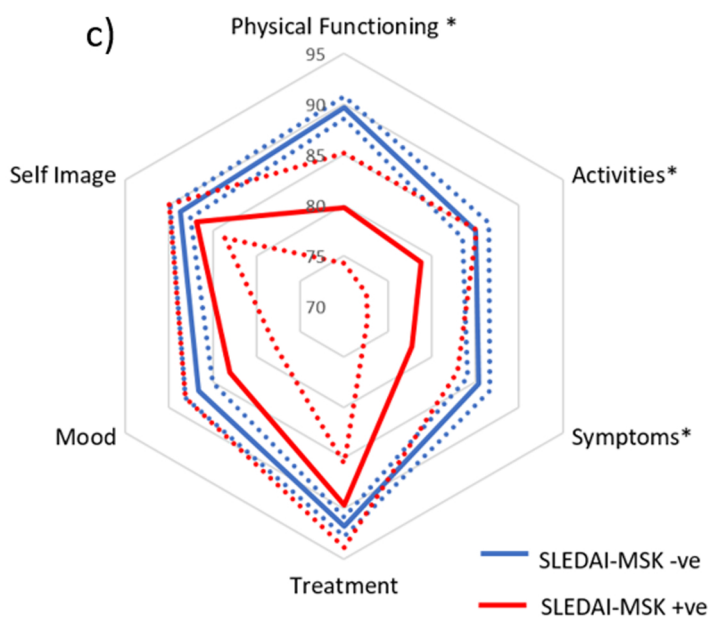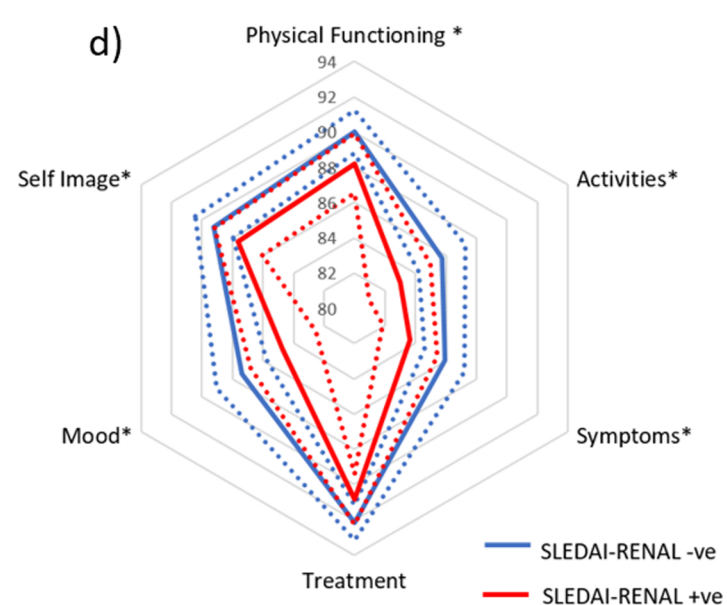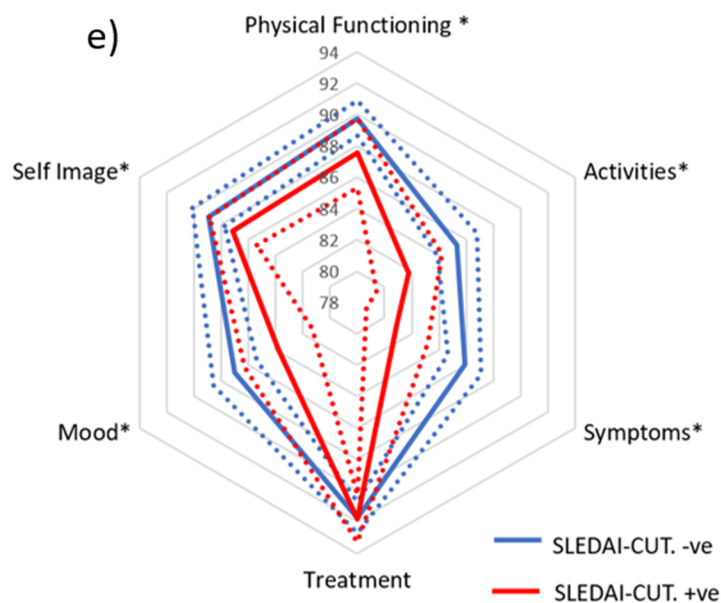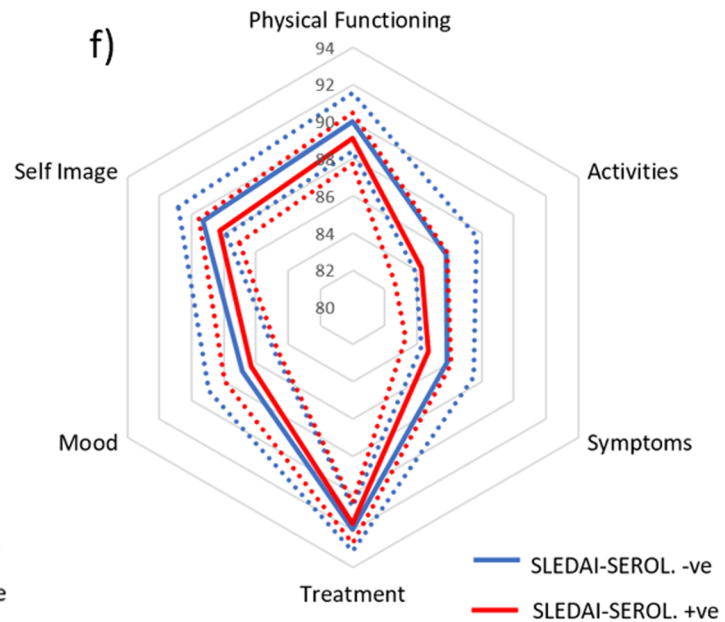

Supplement: Supplementary file 3 — Additional file 3: Figure S3. Radar charts showing mean SLEQOL scores with 95% confidence interval according to organ specific disease activity. SLEDAI = systemic lupus erythematosus disease activity index -2K, CNS = central nervous system, VAS = vasculitis, MSK = musculoskeletal, CUT = cutaneous, SEROL = serological. [file 13075_2020_2095_MOESM3_ESM.pdf]

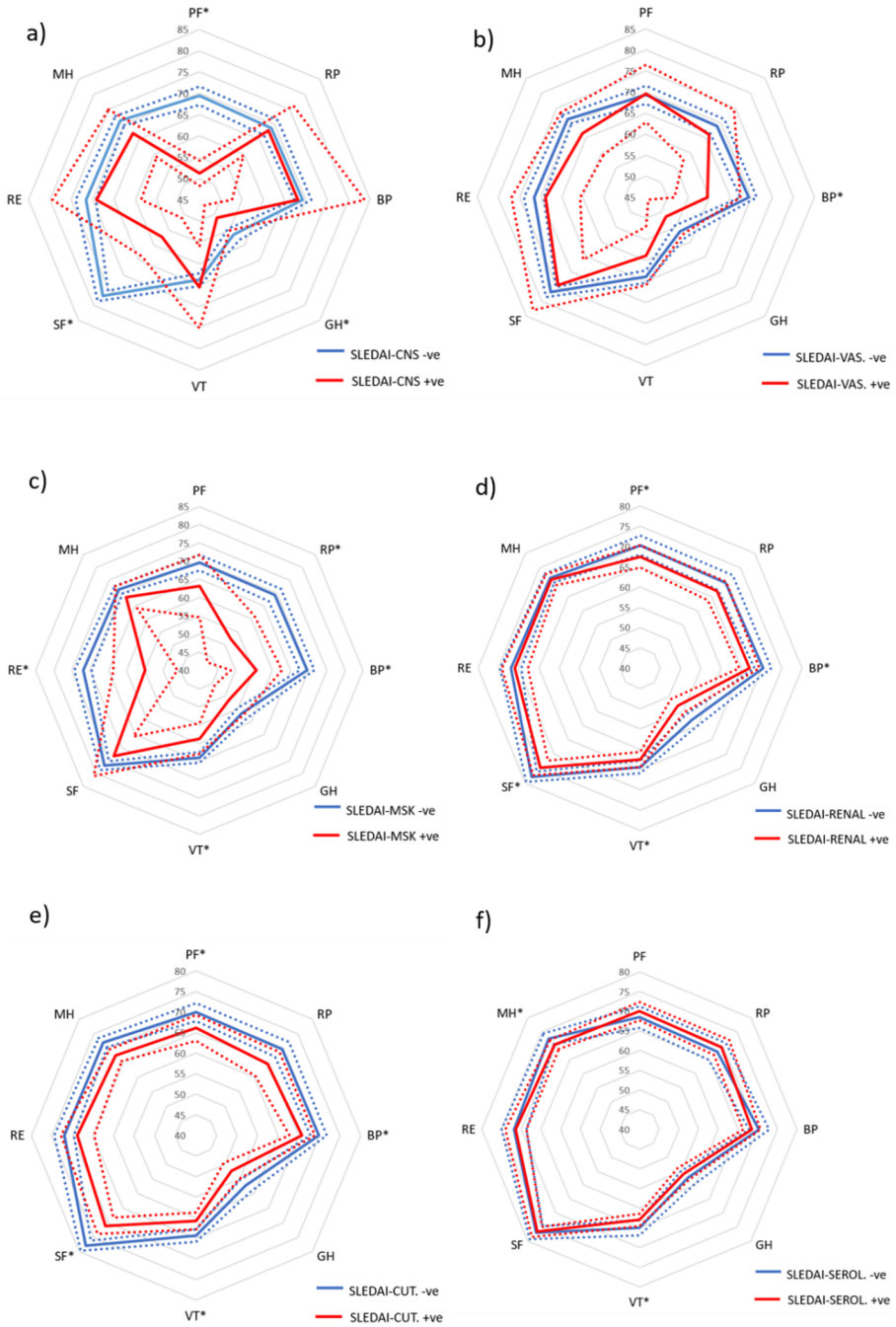

Supplement: Supplementary file 4 — Additional file 4: Figure S4. Radar charts showing mean SF36 scores with 95%confidence interval according to organ specific disease activity. SLEDAI = systemic lupus erythematosus disease activity index-2K, CNS = central nervous system, VAS = vasculitis, MSK = musculoskeletal, CUT = cutaneous, SEROL = serological. [file 13075_2020_2095_MOESM4_ESM.pdf]
