# Supplementary material for: Comparison of performance of specific (SLEQOL) and generic (SF36) health-related quality of life questionnaires and their associations with disease status of systemic lupus erythematosus: a longitudinal study
Source: Arthritis Res Ther. 2020 Jan 10;22:8. doi: 10.1186/s13075-020-2095-4 (PMC6954627; doi:10.1186/s13075-020-2095-4)
Supplement: Supplementary file 5 — Additional file 5: Table S1. Summary of time-adjusted mean scores of health related quality of life indicators. [file 13075_2020_2095_MOESM5_ESM.docx]

**Supplementary Table 1** – Summary of time-adjusted mean scores of health related quality of life indicators

| **TAM SLEQOL Survey scores** | *median [IQR] (range)* | |  |
| --- | --- | --- | --- |
| Total | 89.8 [81.7, 94.9] (1, 100) | |  |
| Physical Functioning (domain 1) | 92.8 [83.8, 97.2] (12, 100) | |  |
| Activities (domain 2) | 89.0 [79.1, 95.1] (0, 100) | |  |
| Symptoms (domain 3) | 88.2 [80.0, 94.0] (0, 100) | |  |
| Treatment (domain 4) | 94.4 [88.0, 98.1] (41.0, 100) | |  |
| Mood (domain 5) | 91.0 [80.2, 97.9] (5.1, 100) | |  |
| Self-Image (domain 6) | 92.4 [81.6, 97.0] (30.3, 100) | |  |
| **TAM SF36 survey scores** | *median [IQR] (range)* | |  |
| Physical Component Summary (PCS) | 46.8 [42.0, 52.1] (17.6, 60.2) | |  |
| Mental Component Summary (MCS) | 49.4 [42.9, 55.0] (20.5, 63.8) | |  |
| Physical Functioning (PF) | 70.7 [55.4, 86.4] (5, 100) | |  |
| Role Physical (RP) | 70.0 [53.1, 87.8] (0, 100) | |  |
| Bodily Pain (BP) | 70.8 [55.1, 84.0] (0, 100) | |  |
| General Health (GH) | 57.1 [41.6, 69.5] (0, 97.8) | |  |
| Vitality (VT) | 64.1 [53.2, 74.1] (10, 98.0) | |  |
| Social Functioning (SF) | 77.9 [63.3, 90.0] (0, 100) | |  |
| Role Emotional (RE) | 73.9 [55.4, 92.8] (0, 100) | |  |
| Mental Health (MH) | 72.1 [61.9, 83.3] (16, 97.5) | |  |
| **GRC status** | |  | |
| Reported improvement at least once*, n (%)* | | 283 (84.0%) | |
| Reported deterioration at least once, *n (%)* | | 133 (39.5%) | |
| Reported no improvement at least once, *n (%)* | | 213 (62.2%) | |
