# Supplementary material for: Comparison of performance of specific (SLEQOL) and generic (SF36) health-related quality of life questionnaires and their associations with disease status of systemic lupus erythematosus: a longitudinal study
Source: Arthritis Res Ther. 2020 Jan 10;22:8. doi: 10.1186/s13075-020-2095-4 (PMC6954627; doi:10.1186/s13075-020-2095-4)
Supplement: Supplementary file 6 — Additional file 6: Table S2. Univariable GEE associations of patient demographics with health-related quality of life surveys. [file 13075_2020_2095_MOESM6_ESM.docx]

**Supplementary Table 2** – Univariable GEE associations of patient demographics with health-related quality of life surveys

| **Demographics** | **SLEQOL** | **SF36-PCS** | **SF36-MCS** |
| --- | --- | --- | --- |
|  | RC* (95% CI),  p-value | RC* (95% CI),  p-value | RC* (95% CI),  p-value |
| Age at enrolment (years) | -0.06 (-0.16,0.04) p=0.2 | -0.17 (-0.23,-0.12) p<0.01 | -0.00 (-0.07,0.06) p=0.9 |
| Age at diagnosis (years) | -0.05 (-0.15,0.05) p=0.3 | -0.17 (-0.23,-0.11) p<0.01 | 0.00 (-0.06,0.07) p=0.9 |
| Disease duration (years) | -0.03 (-0.19,0.12)  p=0.7 | -0.02 (-0.13,0.08)  p=0.7 | -0.02 (-0.12,0.08)  p=0.7 |
| Study duration (years) | 0.60 (-0.73,1.94)  p=0.4 | 0.45 (-0.24,1.15)  p=0.2 | 1.30 (0.52,2.07) p<0.01 |
| Female | 0.80 (-7.83,6.23)  p=0.8 | -0.50 (-5.91,4.90) p=0.9 | -0.08 (-4.32,4.16)  p=0.9 |
| SLE family history | -1.68 (-6.32,2.95)  p=0.5 | -0.85 (-3.45,1.76)  p=0.5 | -1.96 (-5.18,1.25)  p=0.2 |
| Education level *(compared to primary)* |  |  |  |
| Secondary | 3.79 (0.48,7.10) p=0.02 | 3.33 (1.46,5.20)  p<0.01 | 1.01 (-1.26,3.28)  p=0.4 |
| Tertiary | 4.10 (1.02,7.18)  p=0.01 | 5.59 (3.82,7.36)  p<0.01 | 2.32 (0.28,4.36)  p=0.03 |

*RC = regression coefficient, i.e., mean change in health-related quality of life survey scores.
