# Supplementary material for: Comparison of performance of specific (SLEQOL) and generic (SF36) health-related quality of life questionnaires and their associations with disease status of systemic lupus erythematosus: a longitudinal study
Source: Arthritis Res Ther. 2020 Jan 10;22:8. doi: 10.1186/s13075-020-2095-4 (PMC6954627; doi:10.1186/s13075-020-2095-4)
Supplement: Supplementary file 7 — Additional file 7: Table S3. Patient characteristics by GRC categories. [file 13075_2020_2095_MOESM7_ESM.docx]

**Supplementary Table 3** – Patient characteristics by GRC categories

|  | **None** | **No change**  **only** | **Improve.**  **only** | **Deterior.**  **only** | **No change**  **or**  **Improve.** | **No change**  **or**  **Deterior.** | **Deterior.**  **or**  **Improve.** | **All**  **3**  **categories** | **p-value** |
| --- | --- | --- | --- | --- | --- | --- | --- | --- | --- |
|  | **(n=17)** | **(n=15)** | **(n=64)** | **(n=6)** | **(n=108)** | **(n=16)** | **(n=37)** | **(n=74)** |  |
| **Demographics** | **median**  **(IQR)** | **median**  **(IQR)** | **Median**  **(IQR)** | **Median**  **(IQR)** | **Median**  **(IQR)** | **Median**  **(IQR)** | **Median**  **(IQR)** | **Median**  **(IQR)** |  |
| Age at enrolment (years) | 29 (23, 38) | 33 (26, 45) | 39 (28, 49) | 38 (23, 57) | 36 (27, 48) | 38 (35, 47) | 39 (30, 49) | 37 (29, 49) | 0.4 |
| Age at diagnosis (years) | 23 (16, 32) | 22 (16, 32) | 30 (21, 38) | 29 (21, 42) | 25 (18, 40) | 31 (22, 39) | 27 (22, 36) | 25 (19, 39) | 0.4 |
| Disease duration at enrol. (years) | 6 (1, 11) | 9 (4, 13) | 7 (2, 10.5) | 5 (2, 16) | 7.5 (4, 13) | 7 (5, 13) | 8 (4, 18) | 8 (5, 13) | 0.24 |
| Study duration (days) | 0 | 364  (182, 1162) | 1162  (389, 1253) | 175  (168, 210) | 1092  (686, 1211) | 687  (501, 1169) | 1218  (1176, 1323) | 1218  (1155, 1337) | <0.001 |
| Total number of visits | 1 | 3 (2, 8) | 7 (3, 8) | 2 (2, 2) | 7 (5, 8) | 4 (4, 8) | 8 (6, 8) | 8 (7, 9) | <0.001 |
|  | **n (%)** | **n (%)** | **n (%)** | **n (%)** | **n (%)** | **n (%)** | **n (%)** | **n (%)** |  |
| Female | 16 (94.1%) | 15 (100%) | 62 (96.9%) | 5 (83.3%) | 102 (94.4%) | 16 (100%) | 37 (100%) | 72 (97.3%) | 0.4 |
| Current smoker at enrolment | 1 (5.9%) | 0 | 1 (1.6%) | 0 | 1 (0.9%) | 0 | 0 | 0 | 0.5 |
| Family history of SLE | 1 (5.9%) | 1 (6.7%) | 4 (6.3%) | 0 | 9 (8.3%) | 0 | 2 (5.4%) | 8 (10.8%) | 0.8 |
| **Education level** |  |  |  |  |  |  |  |  | 0.09 |
| Primary | 1 (5.9%) | 4 (26.7%) | 14 (22.6%) | 2 (33.3%) | 22 (20.4%) | 3 (18.8%) | 13 (35.1%) | 24 (32.4%) |  |
| Secondary | 3 (18%) | 3 (20.0%) | 25 (40.3%) | 1 (16.7%) | 32 (29.6%) | 3 (18.8%) | 6 (16.2%) | 23 (31.1%) |  |
| Tertiary | 13 (77%) | 8 (53.3%) | 23 (37.1%) | 3 (50.0%) | 54 (50.0%) | 10 (62.5%) | 18 (48.6%) | 27 (36.5%) |  |
| **Medications use** |  |  |  |  |  |  |  |  |  |
| Prednisolone ever | 17 (100%) | 15 (100%) | 60 (93.8%) | 6 (100%) | 98 (90.7%) | 16 (100%) | 35 (94.6%) | 72 (97.3%) | 0.4 |
| TAM Prednisolone dose, *median (IQR)* | 10 (5, 20) | 5 (2.6, 8.7) | 5 (2.8, 7.8) | 19.4 (8.1, 28) | 5.7 (3.2, 9.3) | 7.3 (5.2, 11) | 7.8 (4.9, 10) | 6.6 (4.7, 8.4) | <0.001 |
| Anti-malarials ever | 9 (53%) | 5 (33.3%) | 23 (35.9%) | 2 (33.3%) | 42 (38.9%) | 5 (31.3%) | 16 (43.2%) | 27 (36.5%) | 0.9 |
| Immunosuppressants ever | 11 (65%) | 10 (66.7%) | 49 (76.6%) | 5 (83.3%) | 91 (84.3%) | 13 (81.3%) | 34 (91.9%) | 71 (95.9%) | 0.004 |
| **Clinical indications** |  |  |  |  |  |  |  |  |  |
| TAM PGA, *median (IQR)* | 1.1 (0.9, 1.3) | 0.5 (0.3, 0.6) | 0.3 (0.3, 0.6) | 0.5 (0.3, 0.9) | 0.3 (0.3, 0.6) | 0.4 (0.3, 0.7) | 0.4 (0.3, 0.7) | 0.4 (0.3, 0.6) | <0.001 |
| TAM SLEDAI-2K, *median (IQR)* | 4.0 (4.0, 6.0) | 4.0 (2.5, 5.0) | 2.5 (0.9, 4.9) | 4.3 (2.0, 10.0) | 3.5 (2.0, 5.7) | 3.6 (2.1, 4.9) | 3.7 (2.2, 5.6) | 3.5 (2.0, 5.7) | 0.2 |
| Organ damage present | 6 (35%) | 6 (40%) | 32 (50%) | 3 (50%) | 49 (53%) | 9 (56%) | 23 (62%) | 44 (59%) | 0.5 |
| Achieved LLDAS ever | 4 (23.5%) | 11 (73.3%) | 53 (82.8%) | 4 (66.7%) | 88 (81.5%) | 14 (87.5%) | 32 (86.5%) | 66 (89.2%) | <0.001 |

Improve. = improvement, Deterior. = Deterioration.
