# Supplementary material for: Comparison of performance of specific (SLEQOL) and generic (SF36) health-related quality of life questionnaires and their associations with disease status of systemic lupus erythematosus: a longitudinal study
Source: Arthritis Res Ther. 2020 Jan 10;22:8. doi: 10.1186/s13075-020-2095-4 (PMC6954627; doi:10.1186/s13075-020-2095-4)
Supplement: Supplementary file 8 — Additional file 8: Table S4. Associations of GRC categories with SLEQOL and SF36 domains. [file 13075_2020_2095_MOESM8_ESM.docx]

**Supplementary Table 4** – Associations of GRC categories with SLEQOL and SF36 domains

|  | **GRC categories** | **Mean Score** | **(95% CI)** | | **Mean Change** | **(95% CI)** | **p-value** |
| --- | --- | --- | --- | --- | --- | --- | --- |
| **SLEQOL** | | | | | | | |
| **Physical Functioning**  **(domain 1)** | No change | 90.59 | (89.20 | 91.99) | 0 |  |  |
|  | Deterioration | 79.22 | (76.23 | 82.21) | -11.38 | (-14.52,-8.23) | <0.01 |
|  | improvement | 91.36 | (90.18 | 92.54) | 0.77 | (-0.56,2.09) | 0.3 |
| **Activities**  **(domain 2)** | No change | 86.21 | (84.44 | 87.98) | 0 |  |  |
|  | Deterioration | 74.15 | (70.93 | 77.37) | -12.06 | (-15.44,-8.69) | <0.01 |
|  | improvement | 86.66 | (85.15 | 88.17) | 0.45 | (-0.84,1.74) | 0.5 |
| **Symptoms**  **(domain 3)** | No change | 86.37 | (84.91 | 87.84) | 0 |  |  |
|  | Deterioration | 76.52 | (74.00 | 79.03) | -9.86 | (-12.33,-7.38) | <0.01 |
|  | improvement | 86.92 | (85.66 | 88.19) | 0.55 | (-0.61,1.70) | 0.4 |
| **Treatment**  **(domain 4)** | No change | 91.23 | (90.00 | 92.45) | 0 |  |  |
|  | Deterioration | 89.14 | (87.33 | 90.95) | -2.08 | (-3.84,-0.32) | 0.02 |
|  | improvement | 92.26 | (91.17 | 93.36) | 1.04 | (0.05,2.03) | 0.04 |
| **Mood**  **(domain 5)** | No change | 88.06 | (86.33 | 89.79) | 0 |  |  |
|  | Deterioration | 77.79 | (74.57 | 81.00) | -10.28 | (-13.38,-7.18) | <0.01 |
|  | improvement | 88.78 | (87.23 | 90.33) | 0.71 | (-0.61,2.04) | 0.3 |
| **Self-Image**  **(domain 6)** | No change | 90.15 | (88.79 | 91.51) | 0 |  |  |
|  | Deterioration | 83.78 | (81.64 | 85.91) | -6.37 | (-8.31,-4.44) | <0.01 |
|  | improvement | 89.49 | (88.25 | 90.72) | -0.66 | (-1.71,0.39) | 0.2 |
| **SF36** | | | | | | | |
| **Physical Function** | No change | 69.50 | (66.81 | 72.19) | 0 |  |  |
|  | Deterioration | 61.44 | (58.04 | 64.84) | -8.06 | (-11.53,-4.59) | <0.01 |
|  | Improvement | 70.99 | (68.61 | 73.38) | 1.5 | (-0.74,3.73) | 0.19 |
| **Role Physical** | No change | 71.14 | (68.38 | 73.90) | 0 |  |  |
|  | Deterioration | 60.28 | (56.49 | 64.06) | -10.86 | (-14.60,-7.13) | <0.01 |
|  | Improvement | 71.35 | (68.88 | 73.82) | 0.21 | (-2.04,2.47) | 0.8 |
| **Bodily Pain** | No change | 70.10 | (67.64 | 72.55) | 0 |  |  |
|  | Deterioration | 56.16 | (53.28 | 59.04) | -13.94 | (-16.94,-10.9) | <0.01 |
|  | Improvement | 72.74 | (70.68 | 74.79) | 2.64 | (0.57,4.72) | 0.01 |
| **General Health** | No change | 55.56 | (53.49 | 57.62) | 0 |  |  |
|  | Deterioration | 46.51 | (44.24 | 48.79) | -9.04 | (-11.20,-6.89) | <0.01 |
|  | Improvement | 59.93 | (57.96 | 61.89) | 4.37 | (2.85,5.89) | <0.01 |
| **Vitality** | No change | 64.06 | (62.23 | 65.89) | 0 |  |  |
|  | Deterioration | 58.36 | (56.15 | 60.57) | -5.71 | (-7.91,-3.51) | <0.01 |
|  | Improvement | 66.14 | (64.52 | 67.75) | 2.07 | (0.67,3.48) | <0.01 |
| **Social Function** | No change | 78.35 | (76.23 | 80.48) | 0 |  |  |
|  | Deterioration | 70.48 | (67.56 | 73.41) | -7.87 | (-10.79,-4.9) | <0.01 |
|  | Improvement | 79.33 | (77.31 | 81.35) | 0.97 | (-0.98,2.92) | 0.3 |
| **Role Emotion** | No change | 73.46 | (70.60 | 76.32) | 0 |  |  |
|  | Deterioration | 64.99 | (60.97 | 69.01) | -8.47 | (-12.79,-4.14) | <0.01 |
|  | Improvement | 74.49 | (72.02 | 76.96) | 1.03 | (-1.36,3.43) | 0.4 |
| **Mental Health** | No change | 72.63 | (70.87 | 74.39) | 0 |  |  |
|  | Deterioration | 66.67 | (64.23 | 69.12) | -5.95 | (-8.34,-3.57) | <0.01 |
|  | Improvement | 73.75 | (72.12 | 75.37) | 1.12 | (-0.30,2.54) | 0.12 |
