# Supplementary material for: Comparison of performance of specific (SLEQOL) and generic (SF36) health-related quality of life questionnaires and their associations with disease status of systemic lupus erythematosus: a longitudinal study
Source: Arthritis Res Ther. 2020 Jan 10;22:8. doi: 10.1186/s13075-020-2095-4 (PMC6954627; doi:10.1186/s13075-020-2095-4)
Supplement: Supplementary file 9 — Additional file 9: Table S5. Summary of statistically significant associations between SLE clinical indicators and HRQoL surveys. [file 13075_2020_2095_MOESM9_ESM.docx]

**Supplementary Table 5 –** Summary of statistically significant associations between SLE clinical indicators and HRQoL surveys

|  | **SLEQOL** | | | | | | |  | **SF36** | | | | | | | | | |
| --- | --- | --- | --- | --- | --- | --- | --- | --- | --- | --- | --- | --- | --- | --- | --- | --- | --- | --- |
|  | **Overall** | **Domain-Specific** | | | | | |  | **Component Summaries** | | **Domain-Specific** | | | | | | | |
|  | **Total** | **D1** | **D2** | **D3** | **D4** | **D5** | **D6** |  | **PCS** | **MCS** | **PF** | **RP** | **BP** | **GH** | **VT** | **SF** | **RE** | **MH** |
| **Clinical Indicators** |  |  |  |  |  |  |  |  |  |  |  |  |  |  |  |  |  |  |
| In LLDAS | ● | ● | ● | ● | ● | ● | ● |  | ● | ● | ● | ● | ● | ● | ● | ● | ● | ● |
| SLEDAI-2K Disease Activity |  |  |  |  |  |  |  |  |  |  |  |  |  |  |  |  |  |  |
| Overall (SLEDAI-2K>4) | ***** | ***** | ***** | ***** | ***** | ***** | ***** |  | ***** | ***** | ***** | ***** | ***** | ***** | ***** | ***** | ***** | ***** |
| Organ specific |  |  |  |  |  |  |  |  |  |  |  |  |  |  |  |  |  |  |
| CNS + | ***** | ***** | ***** | ***** |  | ***** |  |  | ***** |  | ***** |  |  |  |  |  |  |  |
| VAS + |  |  |  |  | ***** |  |  |  |  |  |  |  |  |  |  |  |  |  |
| MSK + | ***** | ***** | ***** | ***** |  |  |  |  |  |  |  |  | ***** |  |  |  |  |  |
| Renal + | ***** | ***** | ***** | ***** |  | ***** | ***** |  | ***** |  | ***** |  | ***** |  | ***** | ***** |  |  |
| Cutaneous + | ***** | ***** | ***** | ***** |  | ***** | ***** |  | ***** |  | ***** |  | ***** |  | ***** | ***** |  |  |
| Serological + |  |  |  |  |  |  |  |  |  |  |  |  |  |  |  |  |  | ***** |
| MM/S flare + | ***** | ***** | ***** | ***** |  | ***** |  |  | ***** |  | ***** | ***** | ***** | ***** | ***** | ***** | ***** |  |
| Organ damage + | ***** | ***** | ***** | ***** |  |  |  |  | ***** |  | ***** | ***** | ***** | ***** |  |  |  |  |

● = positive association (i.e. improved HRQoL) with p<0.05; *****=negative association (i.e., worsen HRQoL) with p<0.05

CNS = Central Nervous System, VAS = Vasculitis, MSK = Musculoskeletal, D1 = Physical Functioning, D2 = Activities, D3 = Symptoms, D4 = Treatment, D5 = Mood, D6 = Self Image.
